# Genomic analysis and temperature-dependent transcriptome profiles of the rhizosphere originating strain *Pseudomonas aeruginosa *M18

**DOI:** 10.1186/1471-2164-12-438

**Published:** 2011-08-31

**Authors:** Da-Qiang Wu, Jing Ye, Hong-Yu Ou, Xue Wei, Xianqing Huang, Ya-Wen He, Yuquan Xu

**Affiliations:** 1 State Key Laboratory of Microbial Metabolism, School of Life Sciences and Biotechnology, Shanghai Jiao Tong University, 800 Dongchuan Road, Shanghai 200240, PR China

## Abstract

**Background:**

Our previously published reports have described an effective biocontrol agent named *Pseudomonas *sp. M18 as its 16S rDNA sequence and several regulator genes share homologous sequences with those of *P. aeruginosa*, but there are several unusual phenotypic features. This study aims to explore its strain specific genomic features and gene expression patterns at different temperatures.

**Results:**

The complete M18 genome is composed of a single chromosome of 6,327,754 base pairs containing 5684 open reading frames. Seven genomic islands, including two novel prophages and five specific non-phage islands were identified besides the conserved *P. aeruginosa *core genome. Each prophage contains a putative chitinase coding gene, and the prophage II contains a *capB *gene encoding a putative cold stress protein. The non-phage genomic islands contain genes responsible for pyoluteorin biosynthesis, environmental substance degradation and type I and III restriction-modification systems. Compared with other *P. aeruginosa *strains, the fewest number (3) of insertion sequences and the most number (3) of clustered regularly interspaced short palindromic repeats in M18 genome may contribute to the relative genome stability. Although the M18 genome is most closely related to that of *P. aeruginosa *strain LESB58, the strain M18 is more susceptible to several antimicrobial agents and easier to be erased in a mouse acute lung infection model than the strain LESB58. The whole M18 transcriptomic analysis indicated that 10.6% of the expressed genes are temperature-dependent, with 22 genes up-regulated at 28°C in three non-phage genomic islands and one prophage but none at 37°C.

**Conclusions:**

The *P. aeruginosa *strain M18 has evolved its specific genomic structures and temperature dependent expression patterns to meet the requirement of its fitness and competitiveness under selective pressures imposed on the strain in rhizosphere niche.

## Background

Previously published work from our group has described an effective biocontrol strain M18 that was isolated from sweet melon rhizosphere in Shanghai suburb in 1996 [1]. This strain has been named as the *Pseudomonas *sp. M18, as its 16S ribosomal DNA sequence shares a high similarity to that of *P. aeruginosa *[1], as well as high sequence identities of several global regulatory genes, including *gacA *[2], *rpoS *[3], *qscR *[4], *rhlI *[5], *lasI *[6] and *rsmA *[7]. However, the strain M18 has developed several unusual features which distinguish it from that of the clinically significant *P. aeruginosa *strains, and indeed are more similar to soil-inhabiting biocontrol *Pseudomonas *strains, such as *P. fluorescence *2-79 [8], Pf-5 [9] and *P. chlororaphis *PCL1391 [10]. Firstly, the predominant phenazine produced by the strain M18 is phenazine-1-carboxylic acid (PCA) rather than pyocyanin (PYO) [11]. PCA is considered to be a major biocontrol compound [12], while PYO is not necessary for fungal killing [13]. It was determined that more PCA could be produced in the strain M18 at 28°C rather than 37°C compared with that produced by the *P. aeruginosa *strain PAO1 [11]. Secondly, the strain M18 is the first strain that was reported to produce two antibiotics, PCA and pyoluteorin (Plt) in one single strain [1]. The production of the two antibiotics of the strain M18 can result in synergistic antifungal activities against several phytopathogens. The biosynthesis gene clusters of two antibiotics share 99% nucleotide sequence identity with the clusters of Liverpool Epidemic Strain *P. aeruginosa *LESB58. However, a 5-base-pair deletion exists in the *pltB *gene of Plt synthetic cluster resulting in a frameshift mutation in the strain LESB58 [14]. Thirdly, several interesting features with regards to the regulatory mechanism of the production of these two antibiotics were found to be quite different from that in *P. aeruginosa*. For example, PCA production is negatively regulated and Plt production is positively regulated by a global regulator named GacA [2]. However, the converse relationship occurs in the strain M18 through another global regulator, RsmA [7]. In a more recent study, we demonstrated the negative effect of the QscR regulator on PCA production, but not on Plt in the strain M18 [4]. Studies have also been conducted on the differential regulation of PCA and Plt production by potential quorum sensing (QS) signaling molecule *N*-acyl homoserine lactones (AHLs) secreted by this strain [5,6]. The interrelationship between the QS *las *and *rhl *system in strain M18 are also different from that in strain *P. aeruginosa *PAO1 [15].

Besides the biocontrol strain M18, several *P. aeruginosa *strains, i.e., 7NSK2 [16], PNA1 [17], NJ-15 [18], PUPa3 [19], which inhabit the rhizosphere niche, have been found to show excellent plant growth promoting feature and effective biocontrol property against various phytopathogens, and are utilized for biopesticide development. The genetically modified strain M18 has been recently developed as a high yield antifungal antibiotic PCA producer [20-22]. However, little information is available on their specific genome structures, transcriptome profiles and virulence activities compared to their clinical counterparts.

A series of complete genomic sequences of *P. aeruginosa *strains that originated from patients [14,23-25] have been published recently, and normal environmental *P. aeruginosa *isolates were chosen to compare their virulence and genomic structures [26,27]. To date, none of *P. aeruginosa *strains that originate from the rhizosphere has been completely sequenced and analyzed in detail. This study aims to delineate the genomic structural differences among various *P. aeruginosa *strains that originate from nosocomial patients and rhizosphere niches, and to improve our general understanding of what kinds of strategies have been adopted by the *P. aeruginosa *genome in response to various environment conditions, especially to different temperatures. In this report, the complete genome of the strain M18 was sequenced and compared with those of previously sequenced nosocomial strains. Furthermore, the antimicrobial agent resistance *in vitro *and the competitive growth ability in a mouse model of acute lung infection *in vivo *were investigated in the two strains of M18 and LESB58. The temperature dependent genome expression pattern based on the M18 genome was finally analyzed at 28°C and 37°C.

## Results and discussion

### Strain M18 is an effective biological control agent

**Figure 1:**
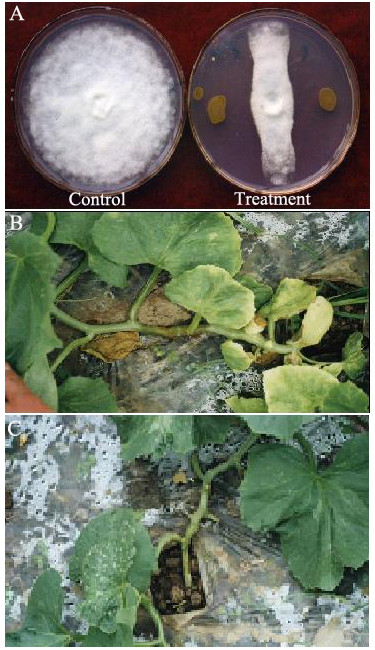
**Biological control ability of *P. aeruginosa *M18**. The growth of the *Mycosphaerella melonis *mycelium plug (inoculated in the center of the PDA plate) was completely inhibited by the metabolites produced by *P. aeruginosa *M18 (A). The initial symptom of fungal disease that appeared on the melon plant (B) and fully recovered after it was sprayed with a suspension of the strain M18 culture (C).

The strain M18 was isolated from the sweet melon rhizosphere that grew in Shanghai suburb in 1996, and proved to have effective biocontrol activity to protect plants against various phytopathogens infection [1]. In this report, we first provide the evidence to show that the secondary metabolites produced from the strain M18 have strong effective inhibitory activity against the growth of the *Mycosphaerella melonis *mycelium plug on a potato dextrose agar (PDA) plate (Figure 1A) and therapeutic anti-fungal effect on the sweat melon plant with full recovery of the plant infected by the *M. melonis *fungus two days after the culture of 10^8 ^CFU/ml of the strain M18 that was sprayed on the infected sweat melon stem (Figure 1B and 1C). We have already found that the strain M18 has a broad spectrum of anti-fungal activities against various phytopathogens, including *Rhizoctonia solani, Sclerotina sclerotiorum, Fusarium oxysporum, Colletotrichum gossypii, Mycosphaerella melonis, Phytophthora capsici, Ralstonia solanacearum *and others. The strain M18 also shows effective inhibiting activity against both the Gram-negative bacterium *Xanthomonas oryzae *pv. *Oryzae*, and the Gram-positive bacterium *Bacillus cereus *(data not shown). Therefore, the unusual features of the strain M18 against various fungal phytopathogens and bacteria prompted us to sequence the complete genome of this strain to ascertain more about the genomic structures, with specific reference to this expression pattern at different temperatures through analysis of microarray-based transcriptome profiles.

### General genomic features and comparative analysis

**Figure 2:**
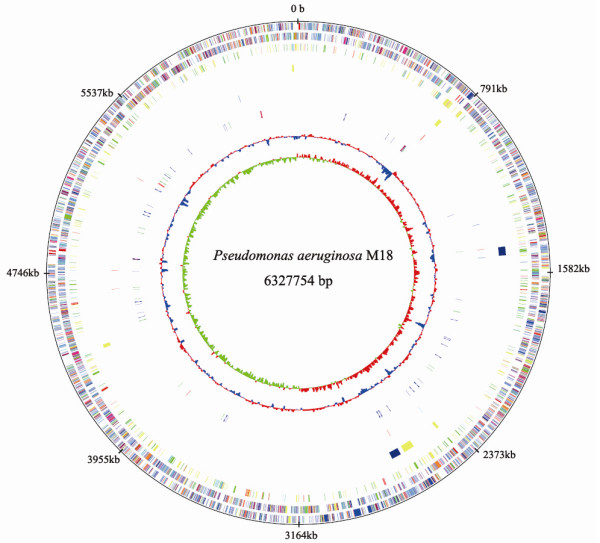
**Circular representation of the *P. aeruginosa *M18 genome**. The chromosome representation was constructed by GenomeViz [75]. Starting from the outermost circle going inwards: the outermost circles indicate coding sequence (CDS) in plus (circle 1) and minus (circle 2) strands are colored by functional categories, as follows: energy production and conversion (orange); cell division and chromosome partitioning (dark orange); amino acid transport and metabolism (maroon); nucleotide transport and metabolism (antique white); carbohydrate transport and metabolism (yellow); coenzyme metabolism (pink); lipid metabolism (tomato); translation, ribosomal structure and biogenesis (peach); transcription (medium purple); DNA replication, recombination and repair (red); cell envelope biogenesis and outer membrane (green); cell motility and secretion (deep pink); posttranslational modification, protein turnover and chaperones (pale green); inorganic ion transport and metabolism (royal blue); secondary metabolites biosynthesis, transport and catabolism (blue); general function prediction only (dodger blue); function unknown (sky blue); signal transduction mechanisms (cyan); intracellular trafficking and secretion (light blue); defense mechanisms (medium purple). Circle 3 indicates temperature-dependent CDSs colored by fold change of transcriptional level, as follows: up-regulated by more than 5-fold (blue); between 5- and 4-fold (light blue); between 4- and 3-fold (green); between 3- and 2-fold (cyan) at 28°C compared with that at 37°C; transcriptional level down-regulated more than 5-fold (red); between 5- and 4-fold (orange); between 4- and 3-fold (pink); between 3- and 2-fold (yellow) at 28°C compared with that at 37°C. Circle 4 indicates genomic islands in M18, colored by prophage (blue) and genomic islands (yellow). Circle 5 represents integrase genes (tomato), transposase genes (light blue) and DNA recombinase genes (dodger blue). Circle 6 represents RNA genes colored by tRNA (blue), rRNA (red), and small RNA (green). Circles 7 and circle 8 represent the GC content and skew, respectively.

The complete genome of the strain M18 was sequenced and assembled as shown in Figure 2. A general comparison of the genomic features of the strain M18 and other sequenced nosocomial *P. aeruginosa *strains is shown in Table 1. The average GC content of the M18 genome is 66.5%, which is remarkably similar to the previously sequenced *P. aeruginosa *strains PAO1, PA14, LESB58 and PA7. The genome of the strain M18 is made of a single chromosome of 6,327,754 bp in size, which is slightly larger than the 6,264,404 bp of strain PAO1 [23], but relatively smaller than the 6,537,648 bp of strain PA14 [24], 6,601,757 bp of LESB58 [14] and 6,588,339 bp of strain PA7 [25]. The M18 genome was annotated to contain 5,684 open reading frames (ORFs) and 80 RNA genes, which represent 89% of the total genomic DNA. Using the mGenomeSubtractor online software [28], we found that the genome similarity among these five *P. aeruginosa *strains is very high; however 93 ORFs in the M18 genome, 109 ORFs in PAO1, 256 ORFs in PA14, 380 ORFs in LESB58 and 929 ORFs in PA7 are strain-specific under the threshold of 0.6 of identity or 0.7 of match length/query length. The strain specific genes contained in the M18 genome are listed in Additional file 1. These results indicated that the available genomes of *P. aeruginosa *strains originating from both clinical and environmental sources contain a highly conserved core genome in large than 90% of ORFs, with several accessory regions in less than 10% of ORFs remarkably varied depending on their different environmental niches (except taxonomic outlier PA7).

**Table 1:** General genomic features of *P.aeruginosa *M18 and other *P. aeruginosa *strains

	M18	PAO1	PA14	LESB58	PA7
Genome size (bp)	6,327,754	6,264,404	6,537,648	6,601,757	6,588,339
GC content (%)	66.5	66.6	66.3	66.3	66.5
Protein coding genes	5,684	5,566	5,892	5,925	6,286
Protein coding density (%)	89	89	89	88	89
RNA genes	80	96	72	102	75
Strain-specific genes (CDS)^a^	93	109	256	380	929
IS	3	12	10	8	15
CDS assigned function^b^					
Translation, ribosomal structure and biogenesis	183	205	205	199	206
Transcription	414	516	537	501	530
DNA replication, recombination and repair	127	160	185	145	235
Cell division and chromosome partitioning	35	34	35	34	37
Post-translational modification, protein turnover, chaperones	186	200	210	201	215
Cell envelope biogenesis, outer membrane	242	265	266	261	260
Cell motility and secretion	150	150	154	149	152
Inorganic ion transport and metabolism	294	376	377	313	355
Signal transduction mechanisms	233	337	345	337	346
Energy production and conversion	304	329	340	330	336
Carbohydrate transport and metabolism	196	252	249	196	250
Amino acid transport and metabolism	474	587	590	490	571
Nucleotide transport and metabolism	107	108	110	104	105
Coenzyme metabolism	178	191	192	210	192
Lipid metabolism	202	244	248	234	245
Secondary metabolites biosynthesis, transport and catabolism	140	205	212	171	198
Function unknown	1,578	1,465	1,706	1,768	2,139

^a ^The strain-specific coding sequences (CDS) were predicted by proteins with low similarity to the other four sequenced *P. aeruginosa *strain genomes (under the expected threshold of 0.6 for identity or 0.7 for match length/query length) using the mGenomeSubtractor [28].

^b ^CDS assigned function was based on the COGs according to RPS-BLAST [66].

**Figure 3:**
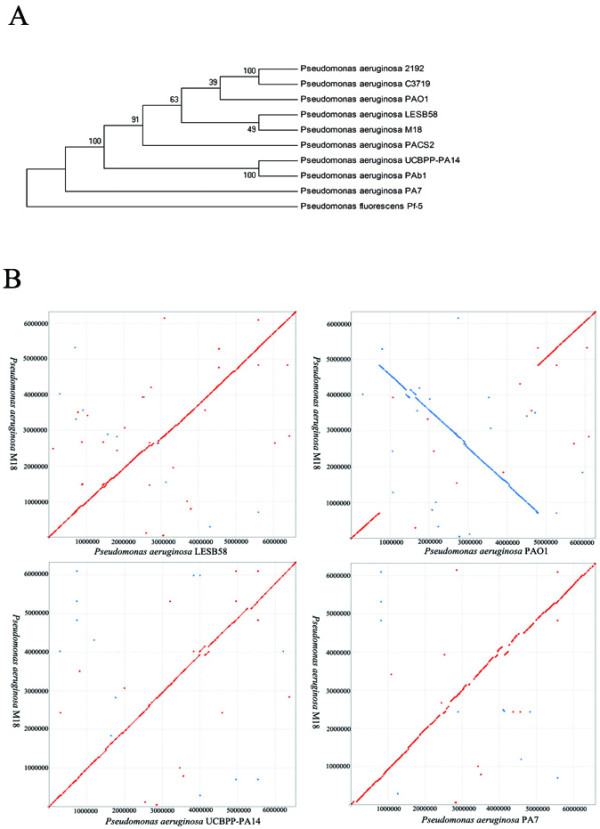
**Genome alignment and phylogenetic analysis of *P. aeruginosa *strains**. A) Total 58 phylogenetically useful genes in *Pseudomonas *spp., which carries more than 10 parsimony informative sites, were selected to construct a phylogenetic tree [27]. The sequences of these strains were aligned by ClustalW [76], and a maximum parsimony (MP) phylogenetic tree was constructed by MEGA 4 [70] with bootstrap 1000. B) Genome alignment of *P. aeruginosa *M18 and other *P. aeruginosa *strains. Line figures depict the results of whole genome alignment results using MUMer [29]. The query genome sequence was the strain M18, and the subject genomes were the strains, LESB58, PAO1, PA14, and PA7. The red lines represent direct alignment, and the blue lines represent reverse alignment, respectively.

Global alignments were performed by the MUMer software [29], and the results showed that the large segment inversion of the chromosome by recombination between the *rrnA *and *rrnB *genes was found in the M18 genome, as well as in other *P. aeruginosa *strains LESB58, PA14 and PA7 (Figure 3B) in comparison with that in PAO1 genome sequenced by the Seattle consortium [23]. Interestingly, the inversion also exists in different PAO1 sublines which are studied and stored in German and American laboratories, and the phenotypes among the PAO1 sublines are also different [30]. This suggests that some physiological consequences occur among the PAO1 sublines and other *P. aeruginosa *strains because of the inversion [27].

The comparison of the M18 genome and the other available *P. aeruginosa *genomes with regards to the functional category breakdown of coding sequences (CDSs) [31] shows a similar distribution in most groups, but the two strains M18 and LESB58 show a greater degree of similarity than others, including a similar number of genes related to DNA replication, recombination and repair, the transport of inorganic ions and amino acids, amino acid metabolism, and the biosynthesis, transport and catabolism of secondary metabolites (Table 1). This suggests that the two strains share a common ancestor as they have the closest relationship in comparison with all other sequenced *P. aeruginosa *strains. The phylogenetic tree was further generated by maximum parsimony (MP) method, using 58 phylogenetic useful genes in *P. aeruginosa *[27]. The result confirmed that the strain M18 is the closest relative to strain LESB58 among the sequence available *P. aeruginosa *strains, as shown in Figure 3A. We also found that nine out of eleven O-antigen loci in the strain M18 share more than 95% similarity to the serotype O6 loci in LESB58 [32], indicating that M18 may carry a similar serotype O6 O-antigen. Based on these sequence data, the strain M18 is now designated as *P. aeruginosa *M18, a new isolate of *P. aeruginosa*.

### Genomic islands and prophages

**Figure 4:**
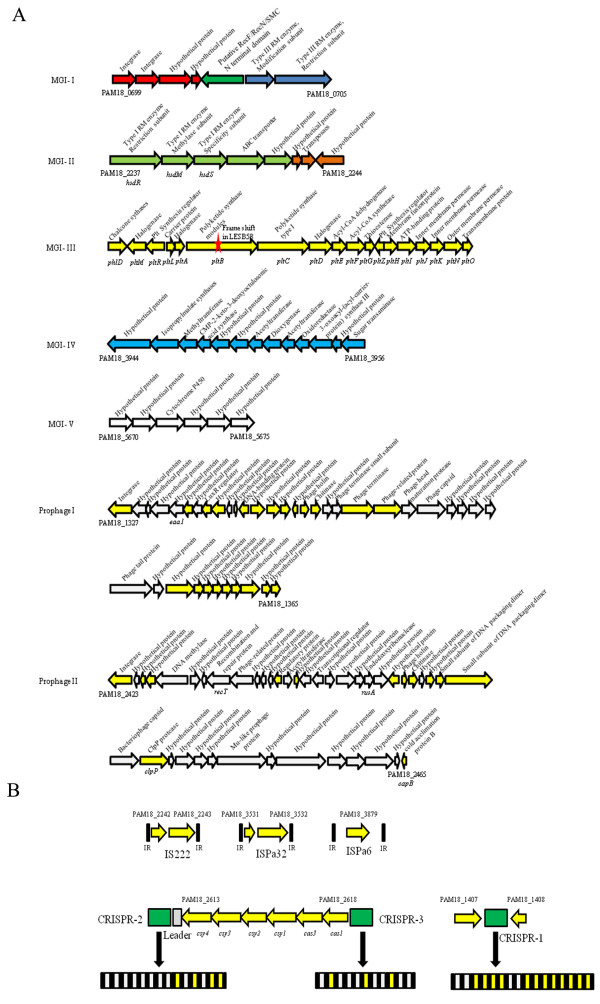
**Genomic islands, prophages, IS and CRISPR loci in strain M18**. A) Genomic islands (GIs) and prophages in the M18 genome. MGI-I contains *mod *and *res *genes coding for type III DNA restriction and modification system (RM) and the genes similar to those in *Pseudomonas putida *GB-1 (red), *Pseudomonas syringae *pv. Tabaci (ATCC11528) (deep green) and *Stenotrophomonas *sp. SKA14 (blue). MGI-II contains genes *hsdR, hsdM and hsdS *encoding for type I DNA RM and the genes similar to those in *Nitrosomonas europaea *(ATCC 19718) (green) and *P. aeruginosa *PA14 (orange). In MGI-III to V, genes similar to *P. aeruginosa *LESB58 (yellow); *P. aeruginosa *PAK (light blue) and the genes which had no significant similarity to other published genes in *P. aeruginosa *(white), The star symbol in MGI-III indicates the frameshift site in the *pltB *gene in LESB58. In prophages, yellow represents genes similar to *P. aeruginosa *LESB58 (share over 30% identity); white represents genes that have no significant similarity to *P. aeruginosa *LESB58. B) The chromosomal organization of insertion sequences (ISs) and clustered, regularly interspaced short palindromic repeats (CRISPRs) with CRISPR-associated (*cas*) genes. In the IS loci, black boxes represent invert repeat (IR) sequences; IS coding genes are located between the two black boxes. The M18 genomic region between CRISPR-2 and CRISPR-3 covered the ORFs from PAM18_2613-PAM18_2618 and name of each are designated. Black boxes indicate the repeat sequence; gray boxes indicate the spacer sequences with over 70% identical sequences to other *P. aeruginosa *genome sequences; white boxes represent the spacers with dissimilar sequences to other *P*. *aeruginosa *strains.

In comparison with other sequenced nosocomial *P. aeruginosa *genomes, the unique regions of genomic plasticity were predicted in the M18 genome, including five specific genomic islands (GIs) designated MGI-I to V and two novel prophages, I and II. The general features of these GIs and prophages in the strain M18 are summarized in Figure 4A and Additional file 2. However, we have not found homologies of the pathogenic islands against mammals such as PAPI-1 [33], PAPI-2 [34], PAGI-5 [35], LESGI-5 [14], in the M18 genome.

To the best of our knowledge, the two GIs, MGI-I and MGI-II are initially found in *P. aeruginosa *M18, and share 38% identity with that in *Pseudomonas putida *GB-1 and 55% with *Nitrosomonas europaea *ATCC 19718, respectively. The GC content of MGI-I (51.7%) and MGI-II (55%) are quite lower than the average GC content (66.5%) of the M18 genome. Difference in the percentage of GC content between the GIs and the core genome is considered as an important marker of horizontal gene transfer, suggesting that these two GIs are associated with recently acquired genetic material. MGI-I contains two genes (PAM18_0699 and PAM18_0700) that code for putative integrases, and MGI-II contains a gene (PAM18_2243) that encodes a putative transposase. MGI-I contains another two genes, *mod *(PAM18_0704) and *res *(PAM18_0705), which encode two subunits of the type III restriction-modification (RM) system. MGI-II contains another three genes, *hsdR *(PAM18_2237), *hsdM *(PAM18_2238) and *hsdS *(PAM18_2239) that code for all the three subunits in the multifunctional type I RM system which quite differ from the two type I RM systems in the PA7 genome. These two restriction systems contained in a single M18 genome can be predicated to assist this strain in digesting foreign genetic material [36], which may explain why *P. aeruginosa *M18 has proven to be recalcitrant to the introduction of foreign DNA and to keep the strain isolated from others within rhizosphere niches.

The GC content of MGI-III (65.6%) is similar to that of the average content in the M18 genome. MGI-III contains a *plt *synthetic gene cluster with its two flanking regulatory genes and transporter gene cluster *(pltMRLABCDEFGZHIJKNO*) [37] and shares 99% nucleotide sequence identity with the LESGI-2 sequence in LESB58, suggesting that the two islands may originate from the same genetic source and remain in the two genomes. However, LESGI-2 contains a frameshift mutation in *pltB *of the *plt *gene cluster [14]. The *plt *synthetic gene cluster is inactivated in strain LESB58, which might the result of the unnecessary Plt production in the nosocomial niche. However, Plt can be produced in the plant-associated pseudomonads such as strain M18 [38] and *P. fluorescens *Pf-5 [39] and plays a crucial anti-fungal function to suppress a variety of plant diseases in rhizosphere niches.

The GC content of MGI-IV (63.1%) and MGI-V (59.5%) is closer to the average content of the M18 genome than those of MGI-I and MGI-II, indicating that the two islands may be not the recently acquired genetic materials. A homologous sequence of MGI-IV was found in *P. aeruginosa *PAK [40]. The MGI-IV contains genes encoding acetyltransferase, 3-demethylubiquinone-9,3-methyltransferase, CMP-2-keto-3- deoxyoctulosonic acid synthetase, phenylpropionate dioxygenase, ring-hydroxylating related dioxygenases, and nucleotide sugar transaminase, which are involved in flagellar glycosylation, secondary metabolite synthesis, and redox reaction in the rhizosphere environment. The MGI-V is a novel genomic island which has never been found in other prokaryotic genomes. The gene (PAM18_5672) in this island encodes a putative cytochrome P450, which can catalyze the oxidation of several metabolic intermediates, such as lipids and steroidal hormones, as well as xenobiotic substances, including drugs and other toxic chemicals [41]. The two genomic islands in the M18 genome may assist the strain M18 to utilize various substances and degrade environmental toxic materials and pollutants in the complex rhizosphere environment.

Two novel prophages named prophage I and II were found in the M18 genome. Each of the prophage contains a putative chitinase gene (PAM18_1344 and PAM18_2447 [42]. Chitinase is a specific degradation enzyme that breaks down glycoside bonds in chitin and is essential for the degradation of chitin, a major component of the fungal cell wall. Besides the three copies of *capB *gene in the M18 genome, an additional copy of *capB *gene was identified in prophage II. The *capB *gene is responsible for encoding a cold shock protein B involving in the adaptation to cold stress in the environment [43]. The two chitinase genes and an additional *capB *gene in the two prophages in the M18 genome suggest that the strain M18 has developed its anti-fungal and cold stress resistant genomic features under the strain-specific environmental selective pressures imposed on it.

### Insertion sequence (IS) and clustered regularly interspaced short palindromic repeats (CRISPR)

A striking feature in the M18 genome is the least number and diversity of 3 IS elements named IS222 (1,227 bp), ISPa32 (1,232 bp) and ISPa6 (1,316 bp) as shown in Figure 4B). However, the other completely sequenced *P. aeruginosa *strains, PAO1, PA14, PA7, and LESB58 carry 12, 10, 15 and 8 IS elements, respectively (Table 1). To our knowledge, the IS elements are postulated to be important drivers of many bacterial genome evolution, such as *Xanthomonas *[44]. In addition to serving as vectors for lateral gene transfer, IS elements can generate other types of genome modification, including rearrangements, inversions and deletions, any of which can increase the instability of the bacterial genome. Therefore, the least number of IS elements in the genome indicates the relative genome stability of the strain M18 in the rhizosphere niches.

As predicted by the CRISPRFinder [45], three CRISPR elements were found in the M18 genome (Figure 4B). CRISPR 1 element is 987 bp in length and has 16 spacers each flanked by a 28 bp direct repeat (DR). There is one single similar CRISPR element in the LESB58 genome, in which six spacers are different from the CRISPR 1 element. The CRISPR 2 element consists of 866 bp with 14 spacers each flanked by 28 bp DR, and the CRISPR3 is 687 bp in length with 11 spacers and 28 bp DR. The CRISPR 2 and 3 sequences are similar to those in the PA14 genome, which only has the two homologies. Furthermore, most of spacers in the CRISPR 2 and 3 elements are quite different from those in other *P. aeruginosa *strains. The strains PAO1 and PA7 only carry some questionable CRISPR elements that have borderline sequence identity. The three CRISPR elements in the M18 genome can be found dispersedly in other sequenced *P. aeruginosa *genomes, indicating that the M18 genome contains the most number of CRISPR elements among the five available complete *P. aeruginosa *genomes. The different spacer sequences in the three CRISPR loci of the strain M18 indicate that M18 has developed specific features to combat various phage invasions in the rhizosphere environment, as spacer sequences are known to be critical for bacteria to resist foreign phage invasion [46,47].

### Biocontrol-related gene clusters with their products

We identified six secondary metabolite biosynthetic gene clusters, which are related to biocontrol activities of the strain M18, and responsible for biosynthesis of two siderophores, hydrogen cyanide, Plt and PCA (Additional file 3). Among them, two gene clusters are responsible for the biosynthesis of two siderophores, including pyoverdine (Pvd) and pyochelin (Pch), respectively, which have been demonstrated to suppress target phytopathogens in the rhizosphere through iron competition [48]. The *hcn *cluster responsible for the production of hydrogen cyanide (HCN) is highly conserved in different *P. aeruginosa *strains. The Plt biosynthesis gene cluster is located in MGI-III and has been described above. The remaining two gene clusters are responsible for PCA biosynthesis. The two *phz *gene clusters in the genome of the strain M18 are highly conserved and share 99% homologous sequences to those in the *P. aeruginosa *PAO1, each of which consists of seven genes (*phzA1B1C1D1E1F1G1 *and *phzA2B2C2D2E2F2G2)*, named *phzA1-G1 *and *phzA2-G2*, respectively [49]. The genes that flank these two clusters are also the same to those of *P. aeruginosa *PAO1. However, there is a 520 bp intergenic region located directly downstream of the *phzA2-G2 *gene cluster between the *phzG2 *gene and the ORF (PAM18_3137) in the strain M18, compared to a shorter 96 bp sequence in the strain PAO1 and a relatively longer 286 bp sequence in the LESB58 genome. All these interval regions are consisted of clustered short repeats, although their function remains unknown.

The predominant phenazine produced in strain M18 is PCA, especially at 28°C, rather than PYO produced in other nosocomial originating *P. aeruginosa *strains [11]. Both PCA and Plt compounds produced by fluorescent *Pseudomonas *species are considered to play an important role in microbial competitiveness and combating plant pathogens [50]. To our knowledge, the strain M18 is the first strain that was reported to produce both PCA and Plt simultaneously, and the synergistic antibiotic effect of PCA and Plt can result in strong activity to protect plants from fungal phytopathogen infections. It has been found that the wild-type strain M18 has much greater effective inhibitory activity against various mycelium plug growths than either the single PCA or Plt null mutants on the PDA plate (data not shown). We purified the two compounds of PCA and Plt individually from liquid culture, and measured the antifungal activities of the single compound and the combinations of various weight ratios of the two compounds against *Rhizoctonia solani *mycelium plug growth on PDA plates, respectively. The 50% value of efficient control (EC_50_) is 8.38 and 58.13 μg/ml for single PCA and Plt, respectively. However, the EC_50 _of the two compound combination at a weight ratio of 10 to 1 at pH 7.0 drops to 0.74 μg/ml only, indicating a significant synergistic effect of PCA and Plt combination on fungal inhibition. The combinations of the two antibiotics may play a crucial role for the strain M18 to protect the plant against different phytopathogen infections.

### Virulence-related genes against mammals

Besides the virulence related genes located in the accessory genome, the LESB58 genome contains all of the 257 genes described for PAO1 with the exception of PA2399 (*pvdD*) and PA1392 [14]. Based on the VFDB database [51], we found that 14 out of 255 virulence genes in the M18 genome are quite different from those of LESB58 after BLASTP searching (Table 2). Firstly, six genes in the M18 genome (i.e., *flgL*, *fliC*, *flaG*, *fliD*, *fliS *and *fliT*) share less than 70% identity with the genes in LESB58, which are involved in the synthesis of flagellum components and related to bacterial motility and biofilm formation. Furthermore, the flagellum of the strain LESB58 belongs to a member of b-type because it lacks flagellar glycosylation, and the flagellum of the strain M18 may belong to the a-type resulted by the flagellar glycosylation function of MGI-IV in the M18 genome. These differences may give rise to different ability of bacterial biofilm formation and induce different mammalian immune system between the two strains. Secondly, five out of 42 pyoverdine synthesis-associated genes, which are involved in the iron uptake and cytotoxic due to its ability to stimulate the production of reactive oxygen species [52], are quite different between the two strains. The gene *pvdS *(PALES28681) is completely absent in the M18 genome. Notably, there are three copies of *fpvA *(encoding the first ferric pyoverdine receptor) and *pvdE *(encoding an ABC transporter) in the LESB58 genome [14], but only one copy of *fpvA *(PAM18_2643) and *pvdE *(PAM18_2644) in the M18 genome; the two genes have only 24.9% and 57.7% sequence identity, respectively, to those in LESB58. Furthermore, *pvdD *(PAM18_2640) and *pvdJ *(PAM18_2641), which are predicted to encode the non-ribosomal peptide synthetase in the pyoverdine synthesis pathway, have only 57.2% and 53.4% sequence identity to those in LESB58, respectively.

**Table 2:** Divergence of virulence genes between *P.aeruginosa *LESB58 and M18.

Divergent virulence gene in LESB58^a^	Gene symbol	Length (AA)	Similar gene in M18	Identity (%)	Match length/Query length
PALES_42341	*flgL*	439	PAM18_3957	67.58	1
PALES_42291	*fliC*	488	PAM18_3943	48.26	1
PALES_42281	*flaG*	123	PAM18_3942	60	0.61
PALES_42271	*fliD*	474	PAM18_3941	42.76	0.98
PALES_42261	*fliS*	126	PAM18_3939	66.39	0.97
PALES_42251	*fliT*	98	PAM18_3938	46.94	1
PALES_49071	*pilA*	154	PAM18_4617	71.61	1
PALES_29051	*pvdE*	550	PAM18_2644	57.71	1
PALES_29021	*pvdE*	550	PAM18_2644	57.71	1
PALES_28991	*pvdE*	550	PAM18_2644	57.71	1
PALES_28971	*pvdD*	4,096	PAM18_2640	57.21	1
PALES_28961	*pvdJ*	1,123	PAM18_2641	53.40	1
PALES_28681	*pvdS*	155	-^b^	-	-
PALES_28981	*fpvA*	819	PAM18_0769	32.27	0.99
PALES_29011	*fpvA*	819	PAM18_0769	32.27	0.99
PALES_29041	*fpvA*	819	PAM18_2643	24.91	0.97
PALES_00751	*ppkA*	1,032	PAM18_0076	98.84	0.58
PALES_27241	*lecA*	122	PAM18_2466	100	0.45

^a ^Virulence genes in *P. aeruginosa *LESB58 were identified based on the VFDB database [51]. Divergent genes were identified by a BLASTP search under the expected level of 75% for identity or 0.7 for match length/query length.

^b ^"-" indicates no homologous was found in the M18 genome.

In the LESB58 genome, the *ppkA *gene (PALES-00751) codes a positive regulator of type VI secretion system [53], and the gene *lecA *(PALES-27241) codes a factor which plays an important role in biofilm formation and adherence to mammalian cells [54]. However, both of the two homologous *ppkA *and *lecA *OFRs (PAM18_2466 and PAM18_0076) in M18 genome are truncated because of a stop codon located in the middle of the two ORFs.

The M18 genome carries a gene cluster responsible for lipopolysaccharide (LPS) O-antigen serotype O6 biosynthesis [32], which shares larger than 90% identity with that in LESB58. Interestingly, the *gmd *gene within the LPS biosynthesis gene cluster coding for GDP-mannose 4,6-dehydratase is a pseudogene in the LESB58 genome [14]. However, the *gmd *gene (PAM18_5576) was found to be a true gene in the M18 genome, although the association with the virulence and O-antigen deficiency remains unclear.

### Attenuated competitive activity of the strain M18 in a mouse model of acute lung infection

**Figure 5:**
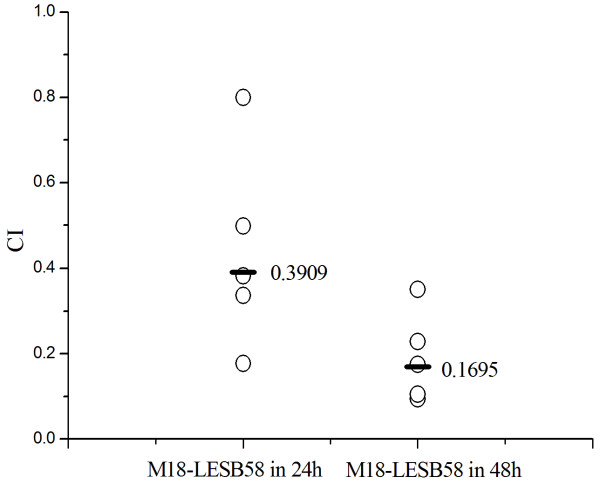
**Competitive index analysis of strains M18 and LESB58 in a mouse model of acute lung infection**. The competitive index (CI) analysis of two strains, M18 and LESB58 at 24 h and 48 h after infection. The CI is defined as the CFU output ratio of the strain M18 in comparison to the strain LESB58 divided by the CFU input ratio of the mixed suspension of both strains. Each circle represents the CI for a single animal in each group. The CI of less than 1 indicates a defect in growth and maintaince of the strain M18 *in vivo *than the strain LESB58. The geometric mean of the CIs for all mice is shown as a solid line.

The different genome structures, especially the different mammalian virulence related genes, prompted us to make a comparison of the infectious activities between the two closely related strains M18 and LESB58 in a mammal model *in vivo*. Thus, we measured the competitive index (CI) of the paired strains to assess their different ability in growth and maintenance in an acute mouse lung infection model (Figure 5). Equal ratio of the two strains was adjusted in phosphate buffered saline (PBS), and the mixture was inoculated into the mouse lung via the nostril. Bacteria were enumerated in the lung tissue at 24 h and 48 h post-infection. The geometric mean CI values were 0.3909 and 0.1695, respectively. The results showed that the strain M18 was easier to be erased than the strain LESB58 in all of the 10 tested mice, and the growth and maintenance abilities of the M18 strain were more attenuated after a prolonged duration of inoculation than LESB58. The preliminary results indicated that the strain LESB58 is more difficult to be erased than the strain M18 in the acute mouse lung infection mode. Further experiments are now being carried out in this laboratory to elucidate the virulence activities of the strain M18 against humans and animal cell lines, and to identify the relationship between the virulence gene expression profiles of the M18 genome in the mouse lung and the responses of mammalian immune system.

### Antimicrobial agent resistance spectrum

The minimal inhibitory concentration (MICs) of antimicrobial agents against the two strains M18 and LESB58 were listed in Table 3. The strain M18 is phenotypically resistant to several antimicrobial agents including penicillins, cephalosporins, chloramphenicol, aminoglycosides and macrolides, which is the same as the reported *P. aeruginosa *strains [55]. The genomic analysis indicated that the strain M18 carries a β-lactamase gene *ampC *(PAM18_0829) and other eight putative β-lactamase genes (i.e., PAM18_0058, PAM18_1371, PAM18_2047, PAM18_2725, PAM18_3247, PAM18_4437, PAM18_5635, and PAM18_5664), which are responsible for encoding enzymes resistant to β-lactam antibiotics [56,57]. Furthermore, the M18 genome contains several gene clusters encoding proteins to constitute efflux pumps, such as *mexAB-oprM *(PAM18_0425-PAM18_0427) [58], *mexCD-oprJ *(PAM18_4693-PAM18_4691) [59], and *mexEF-oprN *(PAM18_2546-PAM18_2544) [60], which result in resistance to many antimicrobial agents including chloramphenicol, aminoglycoside, macrolides and rifampicin. Except the aztreonam, the strain M18 is susceptible to most of the clinically applied antimicrobial agents against *P. aeruginosa*, including third-generation cephalosporins (ceftazidime, ceftriaxone), carbapenems (imipenem, meropenem), monobactam (aztreonam), polymyxin (colistin) and tobramycin. However, comparing with the strain LESB58, the strain M18 is slightly more susceptible to imipenem and meropenem.

**Table 3:** Minimal inhibitory concentrations (MIC) of selected antimicrobial agents for *P.aeruginosa *M18 and LESB58.

Class	Antimicrobial agents	MIC (μg/mL) M18 LESB58	
Penicillins	Ampicillin	512	512
	Carbenicillin	256	256
Cephalosporins	Cefotaxime	256	256
	Cefminox	256	256
	Cefoxitin	256	256
	Ceftazidime	2	2
	Ceftriaxone	8	8
Carbapebems	Imipenem	1	2
	Meropenem	2	4
Monobactam	Aztreonam	64	64
Aminoglycoside	Kanamycin	16	512
	Gentamycin	2	8
	Tobramycin	0.5	0.5
	Spectinomycin	256	256
Polymyxins	Colistin	8	8
Tetracyclines	Tetracycline	16	32
Macrolides	Roxithromycin	32	32
Chloramphenicol	Chloramphenicol	64	64
Rifampicines	Rifampicin	128	128
Quinolones	Ofloxacin	1	4

Interestingly, compared with *P. aeruginosa *LESB58, the strain M18 is more susceptible to several antimicrobial agents, such as gentamycin, kanamycin and ofloxacin. Quinolone resistance is usually present in *P. aeruginosa *strains because of two point mutations in *gyrA *and *parC *in the genome, respectively [61]. The lack of the two point mutations in the M18 genome can be postulated to responsible for quinolone susceptibility of the strain M18. Therefore, the results suggest that the rhizosphere isolate M18 has not evolved specific mechanisms including point mutation or acquisition resistant plasmid to resist antimicrobial agents, unlike nosocomial *P. aeruginosa *strains that face lethal antimicrobial agents stress.

### Genomic microarray based transcriptome analysis

Measurement of gene expression levels at two temperatures, 28°C and 37°C, was performed using an oligonucleotide Agilent microarray based on the M18 genome sequence to specify the temperature-dependent expression profiles of this strain. We selected 28°C as a temperature representative for the rhizosphere niches and 37°C for the human body. The results from the temperature-dependent transcriptome analysis were consistent with our previously published data that the expression of *phzM*, *ptsP *and *lasI *gene is up-regulated at 37°C [11]. The comparative analysis of the M18 genome expressional profiles at 28°C and 37°C indicated that the expression levels of a total of 605 genes at 28°C are regulated over two-fold that at 37°C. It shows that a total of 10.6% of the expressed genes in M18 genome are temperature-dependent (Additional file 4).

In comparison with gene expression level at 37°C, the transcriptional expression levels of 277 genes are up-regulated by approximately two-fold at 28°C, including 151 genes with known functions and 126 functions unknown genes. Among these, 77 function known genes are sorted into nine gene families (Table 4). Surprisingly, 24 of these genes are contained in the M18 GIs and prophage, of which eight genes are located in prophage I, two genes are involved in the type I restriction-modification system in MGI-II, seven genes are related to the pyoluteorin transporter in MGI-III, and seven genes in MGI-V are associated with the synthesis of cytochrome P450. We also found that other up-regulated operons are involved in energy production and the conversion of TCA cycle-related oxidative phosphorylation, aromatic amino acid and benzonate degradation, and phosphate uptake. Besides the operons, a potential phenazine modifying enzyme, *phzH*, a gene that encoded quorum sensing (QS) signal molecule *N*-butanoyl-L-homoserine lactone synthetase, *rhlI*, and a gene that encoded the QS repressor, *qscR*, are promoted at 28°C. The other genes, which are not located in these operons, could be assigned to various functions, including phage-related, FeS cluster assembly, glyoxylate metabolism, benzonate degradation, copper resistance, and pentose phosphate. Hence, the expression levels of these genes involved in antibiotics production, foreign genetic material prevention and nutrient substance degradation are up-regulated at the rhizosphere temperature 28°C, which may give M18 an advantage to resistance the rhizosphere environmental stresses.

**Table 4:** Transcriptomic analysis of *P.aeruginosa *M18 at 28°C and 37°C

Gene family or gene location	Gene ID or name	Fold change
Up-regulated gene at 28°C		
Prophage I	PAM18_1347, PAM18_1349-1350, PAM18_1352-1355	1.8-3.4
MGI II	*hsdR*, *hsdM*	1.8-2.4
MGI III	*pltR*, *pltZ*, *pltHIJNO*	1.4-2.7
MGI V	PAM18_5670-PAM18_5675	2.8-5.4
Phage related	PAM18_0614, PAM18_0616-617, PAM18_0619, PAM18_0620, PAM18_0622-0625, PAM18_0626, PAM18_0627	2.3-8.4
TCA cycle	PAM18_1548-PAM1550	2.1-2.2
Oxidative phosphorylation	*cyoC*, *cyoA*, *napE*, *napF*, *napD*, *napABC*	1.5-2.7
FeS cluster assembly	*iscS*, *iscU*, *iscA*	1.8-2.2
Glyoxylate metabolism	*fdnI*, *fdnH*, *fdnG*	1.6-1.6
Benzonate degradation	*amiE*, PAM18_1600, *amiC*, *amiR*, PAM18_1603, *bkdB*, *bkdA1*, *bkdA2*	1.7-4.5
Phosphate uptake	PAM18_1584, *phnE*, PAM18_1586, *phnJ, phoQ, phoP*	1.6-2.7
Copper resistance	*pcoA, pcoB*	1.4-1.5
Pentose phosphate	*rbsK, rbsR, rbsA, rbsB*	1.6-1.9
Regulatory proteins	*rhlI, qscR*, PAM18_0792, PAM18_0975, PAM18_3016, PAM18_0807, PAM18_3383	2.0-6.1
Down-regulated genes at 28°C		
Type III secretion system	*exoT*, *exsD*, PAM18_0335, *exsC*, *popD*, *popB*, *pcrH*, *pcrV*	2.1-2.8
Type II secretion system	*xcpP*, *xcpR*, *xcpT, secY*	1.6-2.7
Protease production and secretion	*lasA*, *aprA*, *aprD*, *aprE*, *aprF, pfpI*, PAM18_0458	2.3-11.4
HCN synthesis	*hcnA*, *hcnB*, *hcnC*	1.5-2.9
Phenazine metabolism and transport	*phzA1-G1*, *phzM*, *phzA2-G2*, *opmD*, *mexI*, *mexH*	1.6-11.6
Fimbrial biogenesis	*cupC1, pilA, pilN*	2.2-3.0
AHL biosynthesis	*lasI*	2.5
Phospholipase C	*plcB*	3.3
Detoxification	PAM18_1269, PAM18_1457, PAM18_1515, PAM18_4192	2.2-4.6
Benzonate degradation	PAM18_0222-0224, PAM18_0250, *phhABC*, *phhR*, *aroP2*, *hpd*, *hmgA*, *antABC*, *catABC*	2.1-57.0
Transportation	PAM18_0605-0606, PAM18_1782-1783, PAM18_1785, PAM18_2831-2832, PAM18_3039, PAM18_3041-3043, PAM18_5348-5350, PAM18_4688, PAM18_1003	2.0-13.3
Sulfur metabolism	PAM18_3206-3207, PAM18_2369-2372	2.1-3.4
Iron uptake	PAM18_4792-4793, PAM18_4818-4819, PAM18_3537, PAM18_3606, PAM18_4448, PAM18_4605	
Amino acid metabolism	*liuA, liuD, liuR*, PAM18_3044-3046, *arcABC *	
Heat shock proteins	*ibpA, dnaK, groEL*	2.1-3.5
Energy production and conversion	*nqrC, nqrE, nuoEGHIJLMN, atpABCDFGH, nirM, nirS, sucABCD, lpdG*, PAM18_3490-3491	1.6-4.1
Ribosomal proteins	*rplABCDEFJKLMNOPQRSWX, rpmCDEHJ, rpsCEFGHJKLMNQRS*	1.5-3.8
Regulatory proteins	PAM18_0249, PAM18_0710, PAM18_0945, PAM18_0966, PAM18_1156, PAM18_1507, PAM18_2031, PAM18_2078, PAM18_2114, PAM18_2280, PAM18_2571, PAM18_2608, PAM18_3035, PAM18_3129, PAM18_3270, PAM18_3440, PAM18_4328, PAM18_5219, PAM18_5273	2.0-3.9

Cells were grown to OD_600 _= 5.0-6.0 (late exponential phase) in LB medium at 28°C and 37°C, respectively. The effects on gene expression were monitored by microarray analysis using agilent GeneChips. Shown are genes that were located in Genomic islands or are member of gene families in two independent experiments.

In comparison with the gene expression levels at 37°C, a total of 328 gene expression levels are down-regulated by approximately two-fold at 28°C; 53 of these genes have unknown functions, and 189 function known genes are sorted into 19 gene families (Table 4), such as the secretion system, transportation, and ribosomal assembly and etc. These results indicated that the basic protein synthesis, metabolism rate and cell growth rate decrease at the low temperature of 28°C compared to 37°C. Strikingly, none of them is located in M18 GIs or prophages. Interestingly, we found that the 16 down-regulated genes are predicted to encode several mammalian virulence-related factors, including three genes predicated to encode HCN synthetase, three encode proteins involved in type II secretion system, eight genes involved in the type III secretion system, and two genes involved in LasA protease precursor and phospholipase C, respectively.

Transcriptome profile analysis based on the M18 genomic microarray at the two different temperatures implies that the M18 genome has developed its specific features for gene expression to meet the requirement for the competitive fitness and survival in rhizosphere niches except the specific genome structure features.

## Conclusions

The strain M18 isolated from the sweet melon rhizosphere in 1996 in Shanghai suburb was named *Pseudomonas *sp. M18 because of its effective biocontrol ability and several phenotypic features that are similar to *Pseudomonas *spp. [1,2,11]. In this study, the complete genome of the strain M18 was sequenced and shown to share a common core genome that is comprised of more than 90% genome sequence homology to all other sequenced genomes of *P. aeruginosa *isolates (except taxonomic outlier PA7). Based on the comparative genomic and phylogenetic analysis of the M18 genome with other *P. aeruginosa*, the strain M18 can now be definitively designated as a new *P. aeruginosa *strain. The results indicated that the core genome diversity of various *P. aeruginosa *strains from either nosocomial or rhizosphere niche is quite small in comparison with that of various *P. fluorescens *strains [62].

However, the various *P. aeruginosa *strains originating from different environment niches differ in accessory genome regions, genome expression profiles, virulence activities and antibiotic resistances. The analysis of complete M18 genome sequence and temperature dependent transcriptional profiles indicated several important features distinguished the strain M18 from other nosocomial *P. aeruginosa *strains. Firstly, the M18 genome contains several specific accessory regions of genomic plasticity which differ significantly from all sequenced nosocomial isolates. This is the first time to sequence completely a rhizosphere originating *P. aeruginosa *strain M18, and take it as a model strain to analysis and detail the possible determinants related to its biocontrol activity and living ability in rhizosphere niche. We found that the M18 genome contains five specific GIs and two novel prophages, which benefit its survival and biocontrol activity in the rhizosphere niches. Furthermore, all mammalian pathogenicity-related GIs and prophages contained in other sequenced *P. aeruginosa *strains [14,33-35] are absent in M18 genome. Secondly, fourteen mammalian virulence-related genes are absent or truncated in M18 genome, and the competitive index analysis of the strains M18 and LESB58 indicated the strain M18 is easier to be erased than that of LESB58 in the acute infection mouse lung model. Comparing with the strain LESB58, the strain M18 is more susceptible to several antibiotics and antimicrobial agents including kanamycin, gentamycin and ofloxacin. Thirdly, the M18 genome based microarray profiles revealed that the strain M18 has developed its specific temperature-dependent gene expression patterns to meet the requirements of survival and thriving in the rhizosphere niche. Especially, we found that none in the GIs expressed higher at 37°C than that at 28°C, but 24 genes in the GIs and prophages are up-regulated at 28°C which is the temperature close to that in rhizosphere niches.

In summary, the rhizosphere originated strain M18 has evolved specific genome structures and the temperature dependent expressional patterns to meet the requirement of fitness and survival under the selective pressure imposed on it. Therefore, the specific interesting features found in M18 genome and transcriptome profiles may provide us several cues to design therapeutic strategies against *P. aeruginosa *infection, and assist us to develop the strain M18 or other rhizosphere originating strains as a safety cell factory for the industrial purposes to produce antifungal compounds or other secondary metabolites through genetically engineered modification.

## Methods

### Bacterial strains, medium, and growth conditions

*P. aeruginosa *M18 was isolated from sweet melon rhizosphere in Shanghai suburb in 1996 and maintained in our laboratory. *P. aeruginosa *LESB58 which is a hyper-virulent strain isolated from CF patient [63], was obtained from Dr. Robert E.W. Hancock. The two strains were typically grown in Luria Bertani (LB) medium at 28°C and 37°C, respectively.

### Genome sequencing

The draft *P. aeruginosa *M18 genome sequence was obtained using GS 20 system (454 Life Science Corporation). Overall, 547,645 high-quality reads were assembled and had an average length that covered 32.9-fold of the genome, and yielded 227 contigs with the 454 assembly tool. Among these, 172 large contigs (> 500 bp) represented 99.18% of the draft sequence. The complete sequence was assembled and finished according to the method described by Zhang [64], and the average error rate of the finished genome was lower than 1 bp in 10 kb.

### Nucleotide sequence accession number

The annotated genome sequence has been deposited into GenBank under accession no. CP002496.

### Genome annotation

Genome annotation of the strain M18 was mainly performed as described previously [64]. In brief, putative coding sequences (CDS) were predicted using Glimmer 3 [65], and the short CDS (< 90 bp) were eliminated. The putative protein function were predicted using BLASTP searches against all the annotated proteins of the *P. aeruginosa *strains, LESB58, PAO1, UCBPP-PA14 and PA7, and the nr (non-redundant protein sequences) database in NCBI based on the most significant matches. Clusters of orthologous groups (COGs) and subcellular localizations for each CDS were predicted using RPS-BLAST [66], PSORTb v.2.0 [67] and LipoP1.0 [68]. Transfer RNAs were predicted by TRNASCAN-SE [69]. Ribosomal RNAs and small RNAs were predicted by BLASTN searches against those of *P. aeruginosa *strains, LESB58, PAO1, UCBPP-PA14 and PA7. ISs were identified by the IS Finder database http://www-is.biotoul.fr/, and CRISPRs were predicted by CRISPRFinder [45].

### Comparative genomics

The *P. aeruginosa *M18 genome sequence was aligned to those of other sequenced *P. aeruginosa *genomes currently at NCBI with the use of MUMmer [29]. The M18 strain-specific CDS were identified with mGenomeSubtractor [28] with 75% identities, or 70% length ratios of matching regions to questionable CDSs.

### Phylogenetic analysis

Nine completely sequenced *P. aeruginosa *genomes were selected to assess the phylogenetic status of M18, while *P. fluorescens *Pf-5 was used as a member of the "out" group. A total of 58 phylogenetically useful genes [27] which carried more than 10 parsimony informative sites, were selected to construct the phylogenetic tree. Then, the nucleotide sequences of 58 PUG genes in these strains were connected, aligned, and constructed to form a maximum parsimony tree using MEGA 4 software [70].

### Genomic island identification

GIs and prophages located in the 3'-end of the annotated tRNA genes were firstly identified using MobilomeFINDER [71]. GIs located in the other sites of M18 genome were identified by mGenomeSubtractor [28]. The large islands that contained more than five CDS were kept. The direct repeat (DR) elements were identified using BLASTN searches. Prophages were identified by manual inspection of phage-related genes.

### Fungal growth inhibition assay

Fungal growth inhibition by *P. aeruginosa *strain M18 was determined by measuring its ability to inhibit the growth of a plug of *Mycosphaerella melonis *mycelium on a potato dextrose agar plate (PDA). Briefly, a plug in 5-mm diameter from the leading edge of *M. melonis *culture grown for 5 days at 28°C on a PDA plate was taken and placed in the center of a fresh PDA plate. The strain M18 cultures grown overnight on a King's medium B (KMB) plate were placed at the position 25 mm away from the mycelium plug on PDA plate. The co-culture plates were incubated at 28°C and scored after 4 or 5 days by measuring the distance between the edges of the bacterial colony and the fungal mycelium plug [72]. Each experiment was repeated twice with three replicates.

### Competitive index analysis in mouse

The acute lung infection mouse model has been previously described [73]. Five mice were used for each experiment. According to the guidelines of ethics committee for animal treatment, adult female BALB/c mice that weighted more than 17 g at the age of 6- to 8-weeks-old were used. The animals were anesthetized using 2.5% avertin by intraperitoneal administration. The bacteria were centrifuged and washed twice with PBS, and the final concentration was adjusted to approximately 1 × 10^8 ^CFU/ml. Equal ratio of the culture of the two strains M18 and LESB58 was mixed for inoculation. With the mice held in an upright position, 10 μl of the bacterial mixture was inoculated twice (20 μl in total) into mice via application into the nostril with a pipette. Mice were sacrificed at 24 h and 48 h post-inoculation and their lungs were used for CFU counts based on the significant colony size differences of the two strains on LB plates, as the colony diameter of M18 colony and LESB58 is 1-2 mm and 0.2-0.5 mm in size after overnight culture, respectively. The two different strain colonies were further verified by colony PCR using *phzG2 *interval region primer, and the PCR products of M18 and LESB58 colonies are 1075 bp and 841 bp in length, respectively. The pair of primer sequences is designed as phzG2Iup: ACTGACACTGAGGTGCGAAAGCG; phzG2Idown: ACCGTATGCGCTTCACTTGACC. The M18/LESB58 CIs *in vivo *were determined according to the method previously described [73].

### Minimum inhibitory concentration (MIC) Determination

The MIC determination method was applied to measure the susceptibility of the two strains of *P. aeruginosa *M18 and LESB58 to a range of antimicrobial agents, as previously described [25]. For each antimicrobial agent, a serial of two-fold diluted agent was generated using an appropriate starting concentration in Müller-hinton broth media. The MIC corresponds to the smallest agent concentration that inhibits the growth of *P. aeruginosa *M18 and LESB58.

### Oligonucleotide microarray experiments

The *P. aeruginosa *M18-specific Agilent Oligonucleotide microarray (Gene Expression Omnibus (GEO) Platform: GPL11372) was designed with the eArray software. For each of 5,684 annotated CDS, a 60-mer oligonucleotide was designed and repeated twice on the microarray. The *P. aeruginosa *strain M18 was cultured overnight in LB medium with 180 rpm at 28°C and 37°C, respectively. The two cultures were inoculated by 100-fold diluted into LB medium for three biological replicates and grown to OD_600 _~5.0-6.0. The total RNA was extracted using TRIzol (Invitrogen) and measured with a BioAnalyzer (Agilent Technologies, Palo Alto, CA, USA). Total RNA was eluted in RNase-free water and purified by QIAGEN RNeasy kit. A total of 2 μg RNA was reverse-transcribed to cDNA by a one-step method. The cDNA was transcribed into RNA by T7 RNA polymerase, modified by aa-UTP at 40°C, before labeling with Cy3 fluorescence dye at 25°C, purified by a QIAGEN RNeasy mini kit, and quantified by the BioAnalyzer. After hybridization, the arrays were washed according to the manufacturer instructions and scanned twice by an Agilent scanner at 100% and 10% photo multiplier tube (PMT). The normalized data was analyzed using an R script [74]. Expression levels of the genes were compared using SBC analysis system (http://sas.ebioservice.com/). Average values of each gene were calculated based on the three biological replicates.

### Oligonucleotide microarray data accession number

The microarray data has been deposited in to GEO under accession no. GSE26518.

## Authors' contributions

YQX, DQW and HYO conceived and designed the experiments. DQW and YWH HYO were responsible for sequencing, finishing and annotating. DQW, YQX, YWH, JY and XW performed experiments and data analysis. YQX, HYO and XQH contributed reagents/materials/analysis tools. YQX, DQW, YWH and HYO wrote the paper. All authors read and approved the final manuscript.

## Supplementary Material

Additional file 1 **Strain specific genes of *P. aeruginosa *M18**. The strain-specific genes were predicted by proteins with low similarity to the other four sequenced *P. aeruginosa *strain genomes (under the expected threshold of 0.6 for identity or 0.7 for match length/query length) using the mGenomeSubtractor [28]. Click here for file

Additional file 2 **General features of genomic islands and prophages in *P. aeruginosa *M18 genome**. The general features of five genomic islands and two prophages were described, including position, ORF numbers, GC content, mobility gene, specific coding genes, homologous regions and predicted function. Click here for file

Additional file 3 **Secondary metabolite biosynthesis gene clusters in *P. aeruginosa *M18**. The gene clusters for producing five secondary metabolites in strain M18 genome. Click here for file

Additional file 4 **Detailed comparative transcriptomic analysis of *P. aeruginosa *M18 at 28°C and 37°C**. the complete lists of genes differentially regulated over two-fold with a *p*-value of 0.05 or lower at the two temperatures of 28°C and 37°C. Click here for file
