# Supplementary material for: Genomic analysis and temperature-dependent transcriptome profiles of the rhizosphere originating strain Pseudomonas aeruginosa M18
Source: BMC Genomics. 2011 Aug 31;12:438. doi: 10.1186/1471-2164-12-438 (PMC3189399; doi:10.1186/1471-2164-12-438)
Supplement: Additional file 2 — General features of genomic islands and prophages in P. aeruginosa M18 genome. The general features of five genomic islands and two prophages were described, including position, ORF numbers, GC content, mobility gene, specific coding genes, homologous regions and predicted function. [file 1471-2164-12-438-S2.PDF]

Additional file 2: General features of genomic islands and prophages in *P. aeruginosa* M18 genome.

Name	Start[a]	End[a]	ORFs	GC%	Mobility gene	Specific coding gene	Similarity to other strains[b]	Predicted function
MGI-I	746314	762519	7	51.68	Transposase	Type III R.M.[c]	38% *P. putida* GB-1	Genome conservation
Prophage I	1453454	1495933	39	61.2	Integrase	Chitinase	38% *P. aeruginosa* LESB58	Fungal killing
MGI-II	2420183	2431619	8	55.02	Integrase	Type I R.M.	55% *N. europaea* ATCC 19718	Genome conservation
MGI-III	2573003	2625581	36	65.62	None	Pyoluteorin synthesis	100% *P. aeruginosa* LESB58	Fungal killing
Prophage II	2646224	2693027	43	62.26	Integrase	Chitinase, capB	No	Fungal killing Cold resistance
MGI-IV	4375171	4391250	13	63.06	None	Glycosylation and metabolite enzymes	100% *P. aeruginosa* PAK	Flagella glycosylation
MGI-V	6297682	6305081	6	59.47	None	Cytochrome p450	None	Substance Metabolism

[a] The start and end sites of each genomic island and prophage were predicted by mGenomeSubtractor and checked manually; [b] Sequence similarity was calculated by BLASTN searches in the NCBI nr database; [c] R.M. indicates the DNA restriction and modification systems.
