# Supplementary figures and images for: Genomic analysis and temperature-dependent transcriptome profiles of the rhizosphere originating strain Pseudomonas aeruginosa M18
Source: BMC Genomics. 2011 Aug 31;12:438. doi: 10.1186/1471-2164-12-438 (PMC3189399; doi:10.1186/1471-2164-12-438)

### Additional file 3: Secondary metabolite biosynthesis gene clusters in *P. aeruginosa* M18

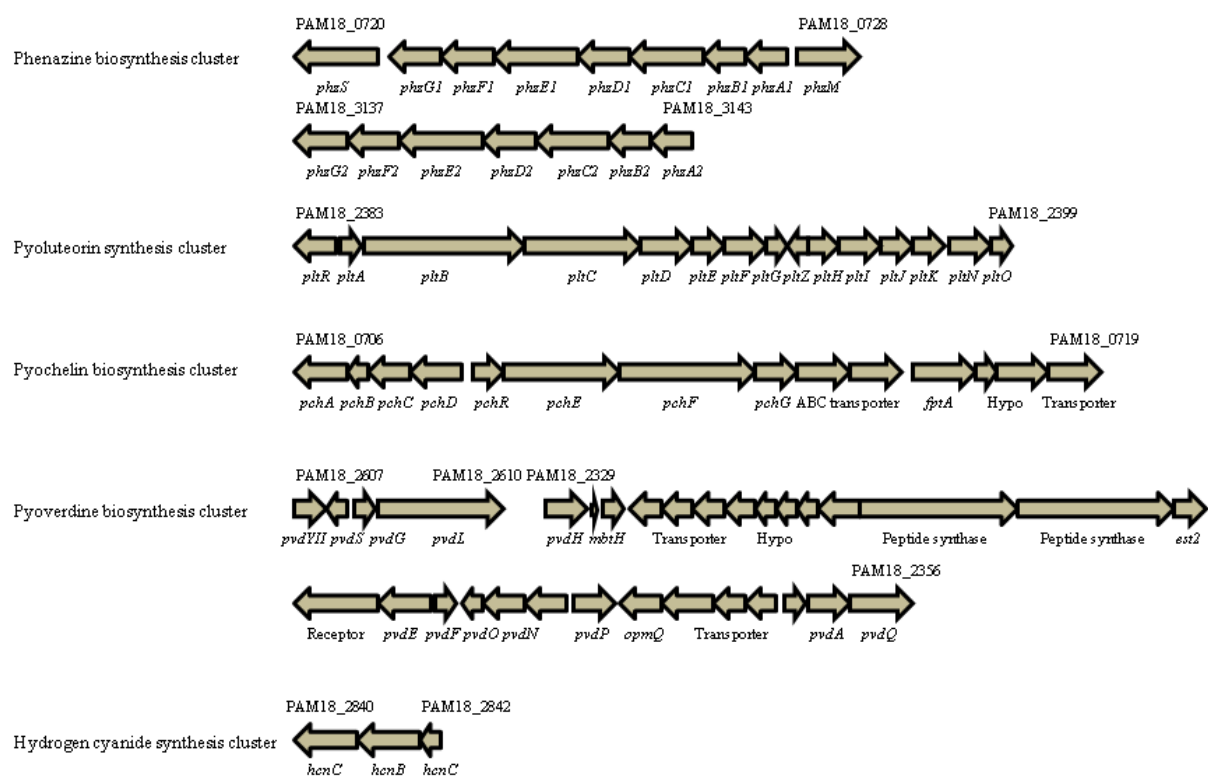

Supplement: Additional file 3 — Secondary metabolite biosynthesis gene clusters in P. aeruginosa M18. The gene clusters for producing five secondary metabolites in strain M18 genome. [file 1471-2164-12-438-S3.PDF]
